# Blood–Brain Barrier, Cell Junctions, and Tumor Microenvironment in Brain Metastases, the Biological Prospects and Dilemma in Therapies

**DOI:** 10.3389/fcell.2021.722917

**Published:** 2021-08-24

**Authors:** Zhiyuan Guan, Hongyu Lan, Xin Cai, Yichi Zhang, Annan Liang, Jin Li

**Affiliations:** State Key Laboratory of Respiratory Disease, National Clinical Research Center for Respiratory Disease, The First Affiliated Hospital of Guangzhou Medical University, Guangzhou, China

**Keywords:** brain metastasis, TME, cell junction, clinical therapy, blood brain barrier

## Abstract

Brain metastasis is the most commonly seen brain malignancy, frequently originating from lung cancer, breast cancer, and melanoma. Brain tumor has its unique cell types, anatomical structures, metabolic constraints, and immune environment, which namely the tumor microenvironment (TME). It has been discovered that the tumor microenvironment can regulate the progression, metastasis of primary tumors, and response to the treatment through the particular cellular and non-cellular components. Brain metastasis tumor cells that penetrate the brain–blood barrier and blood–cerebrospinal fluid barrier to alter the function of cell junctions would lead to different tumor microenvironments. Emerging evidence implies that these tumor microenvironment components would be involved in mechanisms of immune activation, tumor hypoxia, antiangiogenesis, etc. Researchers have applied various therapeutic strategies to inhibit brain metastasis, such as the combination of brain radiotherapy, immune checkpoint inhibitors, and monoclonal antibodies. Unfortunately, they hardly access effective treatment. Meanwhile, most clinical trials of target therapy patients with brain metastasis are always excluded. In this review, we summarized the clinical treatment of brain metastasis in recent years, as well as their influence and mechanisms underlying the differences between the composition of tumor microenvironments in the primary tumor and brain metastasis. We also look forward into the feasibility and superiority of tumor microenvironment-targeted therapies in the future, which may help to improve the strategy of brain metastasis treatment.

## Introduction

In modern society, brain metastasis is a very serious problem affecting public health. It is estimated that about 20% of cancer patients would develop brain metastasis (Bertolini et al., 2015; Achrol et al., 2019; Lah et al., 2020), which is a significant cause of cancer death. Theoretically, any subtype of cancer can metastasize to the brain. The most commonly seen brain metastasis is from the lung cancer, breast cancer, or melanoma. Cancers from the testis, kidney, colon, rectum, and thyroid have a relatively low incidence of brain metastasis (Bertolini et al., 2015; Ostrom et al., 2018). Different cancer types that have metastasized to the brain may cause diverse focal neurologic symptoms and cognitive dysfunction and result in poor life quality ofall patients (Noh and Walbert, 2018). The average survival time of untreated patients with brain metastasis was less than 2 months (Lah et al., 2020). Even with existing treatments, patients with brain metastasis achieve only a median survival time of about 5 months (Cagney et al., 2017).

## Significance of TME in Tumor Biological

A tumor is organ-like structure. It contains not only large amounts of malignant cells but also components from the surrounding environment, such as blood vessels, extracellular matrix, endothelial cells, immune cells, fibroblasts, cytokines and exosomes. They are collectively known as the tumor microenvironment (Balkwill et al., 2012; Denton et al., 2018). Investigation on the tumor microenvironment is crucial for understanding tumor progression mechanisms and discovering therapeutic targets. Tumor cells can change the microenvironment by secreting signal molecules to induce drug resistance and promote tumor angiogenesis (Korneev et al., 2017; Wu and Dai, 2017). The components of the tumor microenvironment, such as tumor-associated macrophages, can also have a feedback effect on tumors and help tumor cells evade immune surveillance (Arneth, 2019). The interaction between the tumor and its microenvironment plays a significant role in tumor progression, invasion, metastasis, and resistance to treatment (Hanahan and Coussens, 2012).

## Significance of TME in Brain Metastasis

The blood–brain barrier and blood–cerebrospinal fluid barrier normally block the inflammatory cells and viruses in the peripheral circulation, which might serve to keep the brain in an immunosuppressive state and help maintain its function (Alexander, 2018). The blood–brain barrier, consisting of endothelial cells, astrocytes, and other components of the central nervous system, is vital to the mammalian brain for its special function of regulating the transportation of molecules into the central nervous system. It also acts as a construction system that contributes to tissue homeostasis and repair (Mastellos et al., 2016). A disease state allows harmful components in circulation to easily penetrate the dysfunctional blood–brain barrier (Montagne et al., 2017; Varatharaj and Galea, 2017; Arvanitis et al., 2020). The formation of the tumor will destroy the integrity of the blood–brain barrier and the blood–cerebrospinal fluid barrier, which will inevitably cause damage to the original environment. This unique condition allows tumors located in the brain to have unique cell types, anatomical structures, metabolic constraints, and immune environments. In addition, because the tumor cells have a huge demand for oxygen and energy, brain metastasis will change the surrounding environment to serve the needs for survival. Brain metastasis not only secretes serpins to inhibit plasmin formation but also recruits astrocytes to promote tumor growth and increase resistance to treatment (Lowery and Yu, 2017). The environment around the tumor is in a state of periodic hypoxia, which can make it insensitive to radiation therapy (RT) (Dewhirst et al., 2008; Michieli, 2009). Brain metastases often provides energy for itself through glycolysis and pentose phosphate pathways. For one thing, this kind of behavior will cause the reduction of nutrients in the surrounding environment. For another thing, acidic substances produced by metabolism will reduce the pH in the surrounding environment and weaken the cytotoxicity of anticancer drugs (Trédan et al., 2007; Lowery and Yu, 2017). Of course, the relationship between brain metastasis and the tumor microenvironment is not as simple as previously described. However, it is clear that the tumor microenvironment plays an important role in the treatment of brain metastasis. In this review, we discuss the molecular regulatory mechanisms of the tumor microenvironment and take this as the point of penetration to summarize the clinical treatments of brain metastasis that have emerged in recent years.

## Blood–Brain Barrier Penetration and Dysfunction of Cell Junctions

The brain is considered as a sanctuary site for metastatic tumor growth, where the integrity of the blood–brain barrier is vital to block the entrance of most tumor cells. The blood–brain barrier consists of the endothelial cells, basal lamina, and astrocyte footplate with surrounding pericytes (Serlin et al., 2015). Between tightly bonded endothelial cells, there are cell junction proteins mainly including the tight junction and gap junction, which act as a highly selective barrier that resists metastasis to the brain. A comprehensive understanding of the underlying mechanism that causes metastasizing tumor cells to break through the cell junction is necessary for the treatment of brain metastasis.

The brain endothelial tight junctions are mostly considered as the fundamental part of the blood–brain barrier which delivers physical support. The connection between proteins forms a tide cell junction preventing brain metastasis. Otherwise, leaking of the blood–brain barrier happens when the tight junction proteins are downregulated or destroyed (Seelbach et al., 2010). For tight junction proteins, ZO and claudin families are highly related (Jia et al., 2014; Godinho-Pereira et al., 2021). As the most known proteins, ZO-1 and claudin-5 give scope to the normal function of tight junctions of the blood–brain barrier (Dejana, 2004). A study of leukemic cells suggests that tumor cells can secrete matrix metalloproteinases 2 and 9 to down regulate the expression of tight junction proteins including ZO-1, claudin-5, and occludin. The degradation of major components of tight junction results in disruption of the blood–brain barrier (Feng et al., 2011). The expression of matrix metalloproteinases 2 and 9 is found in primary tumors and upregulated in brain metastasis (Arnold et al., 1999). It suggests that the matrix metalloproteinase might get involved in the whole process of metastasis and especially favor tumor cells penetrating the blood–brain barrier (Mendes et al., 2005).

Protocadherin 7 (PCDH7) directly interacts with Cx43 to assemble functional gap junctions between cancer cells and astrocytes. Then brain metastasis tumor cells transport the cGMP and Ca^2+^ to astrocytes through the junction (Giaume et al., 2010). Therefore, the key to cell communication is gap junction. Establishment of carcinoma–astrocyte gap junction disrupts the integrity of the blood–brain barrier. The astrocyte–carcinoma surface not only trigger astrocyte cytokine releasing, most of which are IFNα, TNFα, TGFα, vascular endothelial growth factor (VEGF), and Ang-2, but also activate STAT1 and NF-κB survival signals in cancer cells (Chen et al., 2016; Zou et al., 2019). Furthermore, the existence of Cx43 might enhance the entry of double-stranded DNA exosomes into the astrocyte and activate the expression of the second massager. It indicates that the gap junction also enhances indirect cell–cell contact that leads to cytokine releasing (Müller et al., 2001; Pozzobon et al., 2016; Kawaguchi et al., 2019). Secreted VEGF and Ang-2 further influenced the blood–brain barrier permeability (Avraham et al., 2014). CX43 expression can be mediated by lncRNAs such as CCRC (lncRNA-cardiac conduction regulatory RNA), which blocked cell communication of the carcinoma–astrocyte gap junction (Berghoff et al., 2016). Gap junction establishment and the tight junction dysfunction simultaneously promote brain metastasis (Stamatovic et al., 2016; Fujimoto et al., 2020; Zheng et al., 2020). In conclusion, the leaking of the tight junction and formation of gap junction facilitates the migration of tumor cells through the blood–brain barrier, which are considered as several therapeutic targets against brain metastasis.

CXCR4 is a chemokine of the CXC family, correlated to the development of metastasis (Pozzobon et al., 2016). Both CXCR4 and its ligand (CXC motif ligand 12) exhibit a high level of expression in tumors metastasizing to the brain (Müller et al., 2001; Pozzobon et al., 2016; Kawaguchi et al., 2019). *In vivo* and *in vitro* experiments show that AMD3100, a CXCR4 antagonist, targets the CXCR4/stromal cell-derived factor-1 axis by competitively binding CXCR4 to inhibit the proliferation and invasion of tumor cells (Phillips et al., 2003; Wang J. et al., 2020). AMD3100 also upregulates the expression of tight junction proteins including ZO-1, occludin, and claudin-5 and downregulates the expression of CXCR4, VEGF, and matrix metalloproteinase-9 (Li et al., 2017). AMD3100 potentially adapts to the therapeutic strategy. However, AMD3100 is not yet approved for clinical trial application to BM patients. The COX-1/COX-2 inhibitor meclofenamate sodium was previously shown to inhibit Cx43 gap junction gating, reducing the cytokine release (Chen et al., 2016). Based on these results, an ongoing clinical trial (NCT02429570) was applied.

## The Crucial Cell Types of TME in Brain Metastasis

### Phenotypic Differentiation and Polarization of TAMs

Tumor cells may have the ability to recruit macrophages and induce functional polarization. The polarization and phenotype differentiation of tumor-associated macrophages (TAMs) are affected by the metabolite lactate. Tumor-associated macrophages have two opposite phenotypes (Mu et al., 2018). M1-like macrophages secrete inflammatory cytokines such as CCL5, CXCL9, and CXCL10, which can recruit and activate T cells, whereas M2-like macrophages secrete cytokines that repel T cells which might promote tumor proliferation and metastasis. Liu’s study revealed the mechanism of lactic acid promoting tumor-associated macrophage phenotypic differentiation to M2 in the tumor microenvironment (Liu et al., 2019). Lactic acid plays an important role in tumor angiogenesis and cell proliferation by activating ERK/STAT3 signaling to induce macrophages into M2 phenotype (Mu et al., 2018). M2-like macrophages can also coordinate tissue repair and promote the reconstruction and recurrence of tumor blood vessels. It will undoubtedly further increase the difficulty to the treatment of brain metastases (Hughes et al., 2015; Mantovani et al., 2017).

Traditionally, M1 subpopulations of microglial cell activation enhance the expression of STAT1, to reactivate immune response restricting tumor growth (Wei et al., 2013). M2 subpopulations can promote tumor-associated macrophagesproliferation and lead to the establishment of an immunosuppressive microenvironment (Schulz et al., 2019). In the original microenvironment in the brain where CD4+ and CD8+ lymphocytes infiltrate, macrophage/microglial and astrocyte activation will respond when the central nervous system is invaded. Meanwhile, after inflammation is stimulated, microglial cells imitate the characteristics of macrophages that migrated from the bone marrow, acquiring markers of M1 or M2 phenotypes. The continuum of polarization states from M1 to M2 phenotypes indicates a better-activated immune reaction (Mantovani et al., 2002). A crooked M1/M2 ratio toward the tumor-inhibiting M1 subgroup would be beneficial to the treatment of metastatic brain tumors. M1 macrophage level increased slightly along with the growth of the proportion of CD8+ T cells to CD4+ T cells and population of NK T cells, inducing more tumor-damaging effects after treatment of RT and antiangiogenesis therapy (Peng et al., 2020). Zhao applied DSF/copper ions, a chelate with Rego treatment, to an osimertinib-resistant H1975 tumor cell strain that had metastasized to the brain. It results in the M2 subpopulation repolarizing to an antitumor M1-like phenotype, producing antitumor cytokines such as TGF-β (Zhao et al., 2021). However, a monocyte–macrophage lineage is described as a heterogeneous immune cell population. The presence of the blood–brain barrier largely restricts the transportation of large molecules, which is a vital challenge for the medication of metastatic brain tumors. As the blood–brain barrier displays leakiness when the tumor grows, some drugs administered intravenously remain at a quite low level in cancer tissues. By contrast, medicine with a high dosage does more harm than good (Quail and Joyce, 2017). In NSCLC, the brain metastasis rate is much higher when EGFR/T790 mutation happens (Guan et al., 2016). Yin applied a liposomal system to penetrate the blood–brain barrier and target drug resistance. The combination medication reverses the M2 phenotype and facilitates the reverse of T790m associated with EGFR-TKI drug resistance (Yin et al., 2020). Experimental treatments *in vivo* indicate that immune reactivation assists immune cells in exerting their capability to kill tumor cells or enhance the function of antitumor cytokines. The pattern of immune cell infiltration mightpredict the prognosis of brain metastasis, respond to cytokines and biological agents.

### Loss Function of Microglia

Due to the exploration of the complicated interplay of components in the tumor microenvironment, the functional classification of M1/M2 is far oversimplified (Davies et al., 2013; Tomaszewski et al., 2019). Macrophages/monocytes in the brain can be of different origins and classified as tissue-resident macrophages and bone marrow-derived macrophages (Davies et al., 2013; Gentek et al., 2014). Microglia are an important part. A recent study has shown that microglia pronouncedly and densely accumulate in the brain metastasis peritumoral region and around areas of necrosis, while they are rare in viable tumor tissue areas and the surrounding normal-appearing central nervous system tissues (Berghoff et al., 2013). Usually, microglia are involved in protecting neural structures on account of their activation, exerting a balanced proliferative effect and antiproliferative effect on tumor cells. However, microglia in metastatic lesions exhibit only a low-level activation and appear to exert few cytotoxic effects. Microglial cells have phagocytic and cytotoxic properties. They can release several factors like nitric oxide and pro-inflammatory cytokines that have antitumor properties. But most activated microglia around the metastatic tumor are not induced to increase the production of nitric oxide (He et al., 2006; Ransohoff and Cardona, 2010). Another study points out that neurotrophin-3, a neurotrophic factor in the nerve growth factor family, with increased expression in brain metastasis, is able to reduce the expression of nitric oxide synthetase mediated by MAP kinase and PI3 kinase signaling pathways and promote the mesenchymal–epithelial transition of breast cancer cells to enhance its proliferation and metastasis ability (Tzeng et al., 2005; Louie et al., 2013). In this way, the inflammatory reaction of microglia is prevented in the brain tumor microenvironment, and cancer cells may promote proliferation and metastasis because of low-dose microglial factors whose cytotoxicity to the cancer is time and dose dependent.

### Interaction of Astrocytes and Metastatic Tumor Cells

Besides microglia, astrocytes encircle and infiltrate into tumor lesions in the brain as well (Lorger and Felding-Habermann, 2010). After access to brain lesions, astrocytes altered their phenotype by upregulating the levels of GFAP and inducing a reactive astrogliosis program (Sofroniew and Vinters, 2010). Under normal circumstances, activated astrocytes reject extravasated cancer cells by releasing plasminogen activators. The plasminogen activator can generate plasmin that mobilizes the pro-apoptotic cytokine FasL to kill the infiltrating cancer cells (Massagué and Obenauf, 2016). However, the activation of astrocytes may also promote metastasis when metastatic cells are established (Fitzgerald et al., 2008; Priego et al., 2018). The direct contact of metastatic cells and active astrocytes may be the reason for the alteration of normal astrocyte’s function. As mentioned above, there are some mutations detected only in brain metastasis. For example, PTEN, an important suppressor, is found to be downregulated after the dissemination of tumor cells to the brain in contrast to the primary tumor cells. It turns out that astrocyte-derived exosomes mediate an intercellular transfer of PTEN-targeting miRNAs to metastatic tumor cells, thus resulting in PTEN loss in brain metastatic tumor cells, which enhanced proliferation and reduced apoptosis (Zhang L. et al., 2015). Besides, gap junctions are also involved in the communication between astrocytes and tumor cells. The primary tumor cells would express protocadherin 7 to favor the assembly of carcinoma–astrocyte gap junctions, which will engage with brain metastatic cancer cells. With these channels, the second messenger cGAMP will be transferred from metastatic cells to astrocytes, activating the STING pathway and production of inflammatory cytokines IFNα and TNFα. And these paracrine signals activate the STAT1 and NF-κB pathways in brain metastasis cells, which support tumor growth and chemoresistance (Seike et al., 2011; Chen et al., 2016).

### Lymphatic System in the Brain

In 2015, functional and classical lymphatic systems have been found in the brain. The lymphoid fluid transports to cervical lymph nodes, which are the intermediate station of brain lymphoid circulation (Iliff et al., 2015; Louveau et al., 2015). Lymphatic tissues surrounding the central nervous system play a role in the clearing of antigen and peripheral immune cells out of the brain. Notably, lymphatic fluid flow in the brain shows that AQP4 supported the rhythmic glymphatic function as the peripheral immune system (Hablitz et al., 2020). Lymphatic drainage of the brain may manage immune surveillance and T-cell-mediated immunity against brain tumors (Song et al., 2020), which leads to unhindered growth of the metastatic tumor and adaptive immune reaction. New research illustrated a new image of the brain immune microenvironment. With the further understanding of lymphatic vessels of the brain, more valuable insights will be provided in the near future.

## Metabolic Complementation: A New Balance of Metabolism in TME

There is an interesting phenomenon that several key elements including hypoxia, as well as metabolism of glucose with the change of lactate and glutamine, form a new balance for tumor cells to adapt to the original chaotic environment, that is, metabolic complementation (Eales et al., 2016). One of the characteristics of tumor cells is their high demand for nutrients, to sustain their demanding anabolic needs and energy production rates. In order to satisfy the need for this characteristic, tumor cells will reshape the tumor microenvironment (Reina-Campos et al., 2017). In conclusion, cancer cells create a new metabolic equilibrium to accommodate increased metabolic demands and adapt to environmental changes like hypoxia and deficient nutrition (Pavlova and Thompson, 2016). In fact, except for the non-cellular component in the tumor microenvironment, the interaction among cells in the tumor microenvironment also promotes the formation of this balance (Eales et al., 2016). Ultimately, along with the unlimited proliferation of tumor cells, the remodeling of the tumor microenvironment is induced by hypoxia, aerobic glycolysis, and acidosis (Roma-Rodrigues et al., 2019). In the whole process of metabolic complementation formation, metabolic transformation among hypoxia-inducible factors (HIFs), glucose, lactate, and glutamine dynamically happens in the tumor microenvironment, and it is the basis of the resistance for radiotherapy, chemotherapy, and other kinds of targeting treatment (Tennant et al., 2010). Next, we will discuss two major incidents participating in the equilibrium.

### Hypoxia in Brain Metastasis

Hypoxia has been recognized as an intricate characteristic of the tumor microenvironment, defined as insufficiency of oxygen. In the tumor microenvironment, there is a chaotic vasculature which is composed of leaky vessels with blind ends and shunts, tending to collapse. The structural characteristics of the vessels determine the low levels of oxygen far below the adjacent normal tissues (Carmeliet and Jain, 2000). In the absence of functional vasculature, the rapid proliferation of tumor cells exhausts insufficient oxygen (Eales et al., 2016). The primary homeostatic processes in the tumor microenvironment are disrupted for the above two main reasons, and hypoxia becomes unavoidable. With the force of hypoxia, the components of the tumor microenvironment are highly selected for malignancy, which is fundamentally governed by Darwinian dynamics (Gillies et al., 2012). As a result, hypoxia-resistant, more malignant tumor cells or those that are more easily transferred for metastasis are selected (Graeber et al., 1996). After selection, tumor cells often lead to an adverse effect on clinical treatments for brain metastasis under hypoxia. Indeed, a hypoxic tumor microenvironment leads to severe radio-resistance and endows tumor cells with resistance to chemotherapy for the characteristics of hypoxic cells (Brown and Wilson, 2004; Zhou et al., 2020). Similarly, the vascular targeting drugs as single agents cannot show an expected therapeutic effect on hypoxia-tolerant or more malignant tumor cells, especially with more intense hypoxia after targeting the vasculature (Ribatti, 2011). Incidentally, the ability of cancer cells to escape from innate and adaptive immunity gets enhanced as well (Riera-Domingo et al., 2020). Anyway, there are good reasons to pay more attention to be paid to the hypoxic tumor microenvironment.

The effects induced by hypoxia are mainly adjusted and controlled by HIFs, the major components of hypoxia signaling pathways. HIFs, a heterodimer, consist of oxygen-sensitive subunit HIF-1α (HIF-1α, HIF-2α, or HIF-3α) and a constitutively expressed HIF-1β subunit (Lee and Simon, 2012). The heterodimer dissociated in normoxic conditions. Then, oxygen-dependent prolyl-4-hydroxylases hydroxylate the proline residues in the HIF-1α subunit. Subsequently, the HIF-1α subunit after hydroxylation combined with an E3 ubiquitin ligase, Von Hippel–Lindau protein, leading to the rapid degradation of the HIF-1α subunits. Except for the proline residues, the asparagine residues of HIF-α subunits are also hydroxylated by factors inhibiting HIFs. Under hypoxia, the activity of prolyl-4-hydroxylases and factors inhibiting HIFs are suppressed, resulting in the stabilization and activity of the HIFs; then HIFs bind to the DNA in the hypoxia response elements to promote gene transcription, which means the activation of the hypoxia signaling pathway is basically governed by HIF stabilization (Eales et al., 2016; Lee J. W. et al., 2019; Tirpe et al., 2019). Though HIF-1α does not always fit the overall effect of hypoxia, it is the major factor that widely affects the tumor microenvironment, including the expression of numerous genes in tumor cell progression and metastasis, upregulation of VEGF, and epithelial–mesenchymal transition (Tirpe et al., 2019).

Hypoxia is present in brain metastasis, but not in the usual way like it is in most tumor cells. As mentioned above, HIFs are involved in most events that affect the tumor microenvironment and treatment resistance. However, the genes associated with hypoxia are repressed in brain metastasis *in vivo*, while overexpression is often detected in primary tumors. HIF-1α protein was detected in most types of human tumors, including the bladder, breast, colon, glial, hepatocellular, ovarian, pancreatic, prostate, and renal tumors, compared to little expression of either protein in most normal tissue (Talks et al., 2000; Wingrove et al., 2019). Therefore, whether the influence of hypoxia in brain metastasis is dominated by HIFs needs further discussion. Yet, non-invasive predictive assays and planar and single-photon emission computed tomographic (SPECT) have detected high radiotracer avidity in a patient with brain metastasis, after the patient accepted intravenous administration of 23I-IAZA, an effective marker for hypoxic cells (Parliament et al., 1992).

### Glucose Metabolism in Brain Metastasis

The brain is the most important organ of the human body. Due to the existence of the blood–brain barrier, other metabolic substrates cannot enter the brain for utilization. Glucose and some amino acids can enter the brain through specific transporters, where they are used as energy supply (Simpson et al., 2007; Mergenthaler et al., 2013). Although the brain is only 2% of its body weight, it uses about 20% of the body’s glucose (Raichle and Gusnard, 2002). The metabolic environment in the brain is specific, which requires that tumors that metastasize to the brain be able to overcome this physical limitation. Because of the rapid proliferation of tumor cells, they tend to be reprogrammed to accommodate to the adaptation (Boroughs and DeBerardinis, 2015). The Warburg effect is the most classical metabolic phenotype in tumor cells. Whether in an aerobic or anaerobic state, tumors generate ATP through glycolysis rather than oxidative phosphorylation (Warburg, 1956). When the surrounding environment can provide sufficient glucose, glycolysis can overcome the disadvantage of low unit productivity and provide enough energy for tumor cells (Cairns et al., 2011). Glucose levels in the stroma of tissues and organs are lower than those in the blood and brain. Once glucose crosses the blood–brain barrier, it is transported to glial cells and neurons through glutamate dehydrogenase 1 (GLUT1) and glutamate dehydrogenase 3 (GLUT3) to maintain the energy requirements of various nerve activities (Simpson et al., 2007). Brain metastasis competitively utilizes glucose in the brain, resulting in a glucose-deficient state in the tumor microenvironment, which induce a certain impact on the physiological function of normal brain tissues.

Tumor cells arising glycolysis not because mitochondria are deficient in oxygen (Weinhouse, 1976; Frezza and Gottlieb, 2009) but because, as mentioned earlier, the tumor microenvironment is in a state of hypoxia, which activates HIF-1. Following by HIF-1 activation, the expression of pyruvate dehydrogenase kinases is increased, thereby inhibiting the oxidative metabolism of mitochondria. Meanwhile, the expression of lactate dehydrogenase is activated. It enabled pyruvate to be converted into lactic acid instead of being utilized in the tricarboxylic acid cycle (Kim et al., 2006). Lactate is not a metabolic waste. When glucose is insufficient, lactate becomes a fuel source for tumors (Leithner et al., 2015; Park et al., 2016). In addition to glucose, some amino acids are also used as energy sources for brain metastasis, where the most in demand is glutamine (Reitzer et al., 1979). The metabolism of glutamine in the brain is performed in a circulating manner. Glutamine produces glutamate under the action of glutaminase, which is an important neurotransmitter. Unexploited glutamine was absorbed by astrocytes and regenerated by adding amino groups (Zong et al., 2016; Lowery and Yu, 2017). Glutamate is catalyzed by glutamate dehydrogenase or aminotransferases to form α-ketoglutarate, which can provide energy for cells through the tricarboxylic acid cycle (Altman et al., 2016). Glutamine can also maintain the non-oxidized pentose phosphate pathway by utilizing gluconeogenesis and ensures adequate synthesis of purine to promote cell proliferation (Chen et al., 2015; Tardito et al., 2015).

Accumulation of acidic metabolites such as lactic acid produced by aerobic glycolysis leads to a decrease in interstitial pH, and this feature is found in many types of tumors (Tannock and Rotin, 1989). Existing studies suggest that ammonia released by tumor cells through glutamine metabolism acts against the surrounding acidic environment, which plays an important role in the survival and growth of cancer cells (Huang et al., 2013). The acidity of the tumor microenvironment can also be seen as an evolutionary selection, with a population of cells with upregulated acid tolerance having more survivability (Gillies et al., 2008). It is now widely believed that high levels of lactate produced by glucose metabolism can inhibit the immune response of tumor cells (Husain et al., 2013), promote angiogenesis in the tumor microenvironment (Mu et al., 2018), and resist the effects of drug therapy (Apicella et al., 2018; Qu et al., 2019). It affects the growth and metastasis of tumors in many aspects.

## The Molecular Mechanisms Involved in the Brain TME

A genomic study has indicated significant heterogeneity between matched primary tumors and brain metastasis in terms of somatic mutations. It reflected that some unique mutations are identified only in brain metastasis (Brastianos et al., 2015). Therefore, it is believed that these kinds of genetic differences are partly responsible for metastasis. For example, the serine/threonine kinase mTOR plays an important role in early metastasis through overexpression of its component Rictor, which involves tumor angiogenesis, recruitment of microglia, and apoptosis of Jurkat T cells and primary T cells in the tumor microenvironment (Zhang et al., 2019). So, carrying out further research on the gene functions may contribute to a better understanding of brain metastasis and effective treatment strategies. Zhang has observed that mTORC2 disruption can inhibit EGFR/T790m-positive tumor cell growth by blocking microglial cells’ recruitment in the brain. Meanwhile, the level of PD-L1 protein reduced in mTORC1 or mTORC2 disrupted the H1975 cell line. Besides the analysis of the activated protein signaling, certain subgroups found from metastases share the same features such that AKT, ERK, ERBB4/HER4, and downstream substrates and mTOR in NSCLC are relatively activated. Breast cancer cells also modify an immune-suppressed environment in the brain by modulating macrophages and leukocytes (Lee H. Y. et al., 2019). Expression of functional p53 in tumor cells is a “brake” of M2 polarization, which would be much functional if anti-PD1 therapy was investigated (Kim S.-S. et al., 2019). Generally, brain tumor cells prefer to modulate the tumor microenvironment to create an immune-suppressed tumor microenvironment via alternation of activation of signaling pathways and expression of proteins to polarize the tumor-associated macrophages toward the tumor-promoting M2 subgroup (Kulkarni et al., 2018). These accumulated researchers conclude that tumor cells’ phenotype is a fundamental regulator of brain metastasis progression.

## The TME-Related Potential Therapeutics and the Dilemma of Brain Metastasis Treatment

The current management for brain metastasis is surgery, radiotherapy and systemic medical therapies. Corticosteroids are often prescribed to decrease edema, minimize side effects and prevent the development of encephalopathy. High-dose and long-term application of corticosteroids may cause characterized adverse effects and even worsen life quality (Achrol et al., 2019). Systemic chemotherapies have limited benefit (Tsakonas et al., 2017; Waks and Winer, 2019) due to tight junctions of the blood brain barrier which constrains drug delivery to brain tumors. With all occasions taken into consideration, medical treatment of brain metastasis faces grand challenges including the presence of the blood–brain barrier, blood–cerebrospinal fluid barrier, and immune-suppressed regulation system, when compared with primary tumors.

### Hypoxia-Associated Targeting Therapy

Radiation therapy is widely considered as the gold-standard first-line treatment for brain metastasis (Lin et al., 2004; Cheng and Hung, 2007). For patient with numerous lesions in brain parenchyma whole-brain RT (WBRT) is chosen as the preferred treatment. After WBRT, overall survival (OS) is significantly increased compared with supportive therapy alone, although the risk of significant cognitive impairment may increase as well (Bowman and Kumthekar, 2018). However, patients who received WBRT are far from reaching satisfactory outcomes. A study showed that most patients with brain metastasis and accepted WBRT for 3 months have intracranial and extracranial progression, which and accepted quality of life (Steinmann et al., 2012). For the improvement of WBRT, the combination of tumor-microenvironment-targeted therapy is necessary.

One of the reasons for the unsatisfied efficacy of RT is that most of the brain metastases exhibit hypoxic features, and they are more resistance to damage by radiation (Luo and Wang, 2019), and the clinical prognosis of patient receiving RT is adversely affected. Furthermore, another study showed that the original HIF-1-negative brain metastasis cells turn positive after surviving radiation, which may be attributed to RT resistance as well (Harada et al., 2012).

**FIGURE 1:**
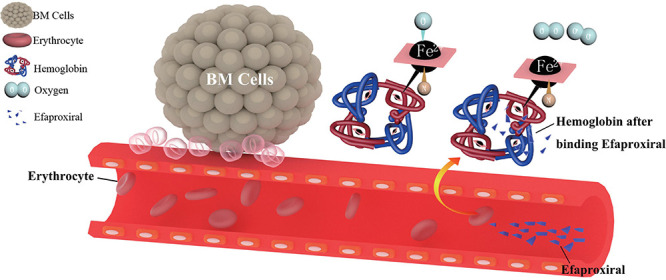
The pharmacological mechanism of efaproxiral. Efaproxiral is an allosteric modifier of hemoglobin. It decreases oxygen affinity by stabilizing the central water cavity of the hemoglobin tetramer (Wireko et al., 1991). With the conformational structure changed, there will be an increase in whole-blood P50 (partial pressure of oxygen which results in 50% hemoglobin saturation) and an increase in the PO_2_ (partial pressure of oxygen) in the tissue, expected to decrease the hypoxic fraction of brain metastasis and increase its sensitivity to RT (Suh, 2004).

Among the ongoing and completed clinical trials, only efaproxiral, a synthetic allosteric modifier of hemoglobin, is designed to improve the hypoxic environment in brain metastasis. As a synthetic allosteric modifier of hemoglobin, efaproxiral emulates the function of naturally occurring allosteric modifier such as 2,3-diphosphoglycerate. It affects the conformational structure of hemoglobin by non-covalently binding in the central water cavity of the hemoglobin tetramer. Therefore, the ferrous ion inside the hemoglobin tends to release oxygen instead of binding, thus reducing the oxygen-binding affinity of hemoglobin. As a result, there will be an acute increase in whole-blood P50 (partial pressure of oxygen which results in 50% hemoglobin saturation) and an increase in the PO_2_ (partial pressure of oxygen) within the tissue, which contribute to the improvement of oxygen level in brain TME (Suh, 2004) (Figure 1). Basically, efaproxiral is used as a radiation sensitizer, a combined treatment to RT. Phases I–III trial data have confirmed the safety profile and dosage of the drug, with the potential benefit of extended survival (Kavanagh et al., 2001; Shaw et al., 2003; Suh et al., 2006).

### Targeting Drugs Related to Glucose Metabolism

Due to the tumor microenvironment exhibited with hypoxic feature, the tumor acquire sufficient energy through specific glucose metabolism patterns. In the tumor microenvironment, acidic metabolites produced by glycolysis can interfere with the effect of chemotherapy drugs (Doherty and Cleveland, 2013; Apicella et al., 2018), thus weaken the killing effect of immune cells on tumors (Brown and Ganapathy, 2020; Wang J. X. et al., 2020). Therefore, it is of great significance to explore anti-tumor drugs that interfere with the process of glucose metabolism. Pyruvate dehydrogenase kinases and lactate dehydrogenase may play an important role in glucose metabolism. In tumor cells, the pyruvate dehydrogenase complex activity is inhibited by pyruvate dehydrogenase kinases, and the pathway of oxidative phosphorylation during the oxidation of pyruvate is blocked. As a result, pyruvate can only conduct glycolysis under the activation of lactate dehydrogenase to produce lactic acid (Zhang S.-L. et al., 2015; Stacpoole, 2017; Woolbright et al., 2019). Dichloroacetic acid is an inhibitor of pyruvate dehydrogenase kinase, which can restore mitochondrial oxidative phosphorylation, block tumor glycolysis, reduce lactic acid production, increase reactive oxygen species in the surrounding environment, and induce tumor cell apoptosis (Garon et al., 2014). Dichloroacetic acid is a small molecule that reaches 100% bioavailability when taken orally (Michelakis et al., 2008). The advantages above enable this molecule to penetrate into the blood-brain barrier and play its role. At present, many clinical drug trials are designed aiming to target the process of glucose metabolism in tumors, but few have been conducted in brain metastasis. According to a phase I clinical trial, oral DCA is well tolerated and safe in patients with glioblastomas, recurrent tumors, and other tumors that have metastasized to the brain (NCT01111097). The application of the drug is feasible, but the specific efficacy of the drug needs further investigation.

### Immune Checkpoint Inhibitors

**FIGURE 2:**
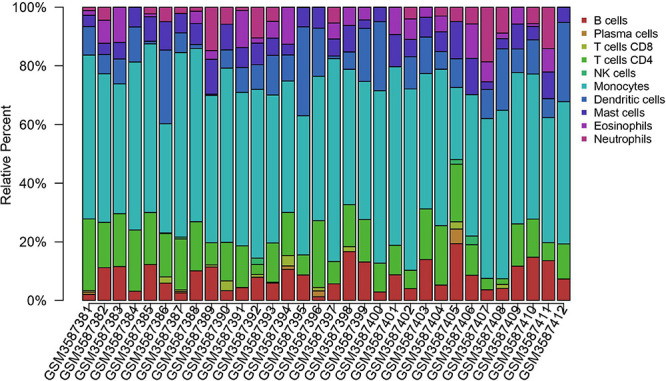
Immune cell infiltration of primary breast cancer and brain metastasis breast cancer. Immune cell infiltration analysis of primary breast cancer and brain metastasis breast cancer using CIBERSORTx. The primary breast cancer samples range from GSM3587381 to GSM3587396; the others are brain metastasis samples. Raw data are downloaded from the GEO database. Data set: GSE125989, https://www.ncbi.nlm.nih.gov/geo/query/acc.cgi?acc=GSE125989. The difference between the primary tumor site and brain metastasis tumor is not statistically significant. More subgroups should be analyzed for a better understanding of the brain metastasis immune microenvironment. Furthermore, further discovery on phenotype and personalized expression is needed to illustrate the immune cell profile.

Inhibitors of immune checkpoints targeting CTLA-4 and PD-1/PD-L1 have been applied in clinical treatment of brain metastasis recently. It was thought that there are no lymphocytes in brain parenchyma in the past; however, tumor-infiltrating lymphocytes (TILs) have been detected in brain metastasis tumor lesions now (Berghoff et al., 2016). More and more research related to immune cells in brain metastasis reveals that brain metastasis owns a unique immune microenvironment different from that of primary tumor or central nervous system tumor. In consideration of new insight into meningeal lymphatic fluid flow, it is no accident that immune therapy will be on a new battlefield to exert its potential effect that has not been fully discovered. There are subtle differences in density between common brain metastasis tumors in the brain. Melanoma has the highest density of TIL, followed by renal cancer and lung cancer (Berghoff et al., 2016). Compared with the primary tumor, brain metastasis lesion was found to have a lower TIL level (Mansfield et al., 2016; Kim R. et al., 2019) and a higher PD-L1 expression level (Mansfield et al., 2016). The different patterns of immune cell infiltration led to diverse responses to immune checkpoint inhibitors. We tried to use a method for quantifying cell fractions from tissue gene expression profiles to analyze the data set GSE125989 (downloaded from the GEO database by estimating relative subsets of RNA transcripts and cell-type identification, CIBERSORT). However, immune infiltration showed that there is no statistical difference in the immune cell types between primary breast cancer and brain metastasis lesion. More specific sequencing data of immune cells derived from tumors are needed to further validate the result (Figure 2). Generally speaking, primary tumors tend to lose their PD-L1/PD-1 expression phenotype when metastasized to the brain (Kluger et al., 2015). The response rate of brain metastasis patients who wasn’t previously treated with single immune checkpoint inhibitor is around 20–33% (Goldberg et al., 2016; Long et al., 2018), slightly higher than that in patients after systematic treatment. The combination of nivolumab and ipilimumab reached an intracranial response rate of 56% (Long et al., 2018).

Tumor-infiltrating lymphocytes emerged as a vital role in antitumor immune response. A recent study showed a high proportion of TIL infiltration in nearly all specimens of brain metastasis and it was irrelevant to TIL density, PD-L1 expression and use of corticosteroid (Berghoff et al., 2016). The brain is not an organ isolated from immune systems. Mechanistic studies have not revealed the main driving factors of the immunosuppressive microenvironment in brain metastasis lesions. Brain edema is a strong inflammatory characteristic correlated with the infiltration of CD8+ lymphocytes and is well recognized. The edema is associated with immune activation in the brain, which involved complicated cell components and cytokines (Berghoff et al., 2016). Inflammatory immune responses in brain metastasis cannot be simply explained as immune activation or suppression. Patients with more significant signs of inflammation in brain metastasis lesions may have better prognosis (Taube et al., 2012; Qian et al., 2016).

The inflammatory cytokine IFN-γ is commonly recognized as the main driving factor meditating PD-L1 and indoleamine-2,3-dioxygenase (IDO) upregulation. PD-L1 on tumor cells preferentially interacts with effector T cells thus providing an advantage for the proliferation of Tregs (Jacobs et al., 2009). IDO is a tryptophan oxidase, which has been proven to contribute to peripheral immune tolerance (Iliff et al., 2015). Brain metastasis lesion of melanoma has been shown to have a high expression of Tregs, as well as IDO and PD-L1 (Spranger et al., 2013). Tregs in brain metastasis tumor has higher expression of CTLA-4 and FoxP3 than blood-derived Tregs, which are related to immune suppression (Jacobs et al., 2009). The accumulation of cytokines further leads to an increase in Treg number (Steindl et al., 2021). Zhao et al. revealed that immune cells in circulation play an important role in establishing a unique immune environment in the brain. The efficacy of the immune checkpoint inhibitor can only be observed when the extracranial tumor was present (Zhao et al., 2021). This brings both challenges and opportunities to checkpoint inhibitor treatment for brain metastasis. Some primary tumor lesions with “hot tumor” characteristic could transform into “cold tumor” when brain metastasis formed. The immune system in the brain seems to have been kept suppressed so as to make it comfortably numb.

### Chimeric Antigen Receptor (CAR)-Based Immunotherapy Therapy

The artificially manipulated CAR is an integral component of CAR T cell which enables recognition of tumor antigens more easily than normal T cells. Well-optimized CAR T cell reduces the “on-target off-tumor” activity of traditional monoclonal antibodies and enhances potential safety (Guo et al., 2018; Priceman et al., 2018; Liu et al., 2020). Meanwhile, CAR-based therapy mitigates antigen escape. Compared with normal T cells, CAR-designed T cells have sustainable antitumor function (Hegde et al., 2019). Normal T-cell immune reaction is observed to initially accentuate functionality followed by an exhausted phenotype when T cell is overstimulated by tumor antigens. Although CAR-T cells have shown remarkable success in B-cell leukemia, it has not shown a promising response to solid tumors. Adaptive changes of the brain tumor microenvironment disturbed the function of T cell/CAR T cell. Tregs played a central regulative role in the adaptive immune suppression reaction in the tumor microenvironment. Once brain tissue damage occurred it triggered Treg amplification in the cervical lymph nodes and brain (Ito et al., 2019). Accompanied with the attendance of cytokines such as TGF-β, IL-10, and IL-2, Tregs are being recruited to tumor microenvironment of brain metastasis lesion. During the process, the function, proliferation, and cytotoxicity of APC, T cells, and NK cells are downregulated (Ghiringhelli et al., 2005; Hawrylowicz and O’Garra, 2005; Zou, 2006; O’Rourke et al., 2017; Ito et al., 2019). To hinder secondary non-CAR T-cell reaction, lymphodepleting chemotherapy is necessary. The 4-1-BB signal is supposed to confer Treg resistance in CAR T cells while CD28 is also required for antitumor efficiency (Suryadevara et al., 2019). 4-1-BB is a co-stimulation signal for T-cell as well as NK cell expansion and activation (Cho et al., 2006; Chester et al., 2018; Claus et al., 2019). Although 4-1-BB agonist therapy can inhibit the differentiation of conventional effector cells into Tregs and suppress the inhibitory effect of Tregs, meanwhile, it would maintain the expansion ability of Tregs (So et al., 2008; Barsoumian et al., 2016). After 4-1-BB was triggered, the cytotoxic effect of NK cell was greatly enhanced, but the negative feedback of 4-1-BB pathway may also inhibit its overactivation, which would attenuate the cytotoxic effect (Baessler et al., 2010; Buechele et al., 2012; Navabi et al., 2015). Recently, 4-1-BB and CD28 are widely designed as signaling domains for improving the effective CAR T-cell antitumor function in brain metastasis (Ahmed et al., 2017; Priceman et al., 2018; Goff et al., 2019). The dynamic balance of effector T cells, Tregs, and memorial T cells, even including NK cells and their interactions, is within the factors considered for CAR-related treatment strategy decisions.

### Other Mono-Antibody Combination Therapies

For breast cancer, ERBB2 (also called HER2 or HER2/neu) is a critical transmembrane tyrosine kinase receptor overexpressed in 15–25% of patients. The patients with an ERBB2+ subtype could obtain an absolute benefit in terms of disease-free survival when treated with trastuzumab, the anti-ERBB2 antibody (Piccart-Gebhart et al., 2005). Trastuzumab, in addition to paclitaxel or docetaxel, and trastuzumab plus vinorelbine have been proven effective for ERBB2-positive breast cancer with brain metastasis (Andersson et al., 2011). In CLEOPATRA, a randomized phase III study demonstrated that patients who received bevacizumab plus trastuzumab and docetaxel gained longer OS. Furthermore, these combined target therapies showed their particular advantages and general safety for ERBB2+ breast cancer brain metastasis (Swain et al., 2013). The anti-VEGF antibody bevacizumab can be applied to monastic cancers as a second-line treatment in phase II/III clinic trials. However, it is still unclear whether anti-VEGF therapy could deliver efficiency in brain metastasis (Besse et al., 2015). Bevacizumab may lead to unacceptable adverse events. Safety results were consistent with observations of proteinuria, hypertension, and even hemorrhage. Rare cases of hypertensive crisis with report encephalopathy and subarachnoid hemorrhage (Besse et al., 2015; Li and Kroetz, 2018). For fear of increasing the risk of adverse events, brain metastasis patients were always excluded. Some trials and observations show contested results that patients with brain metastasis can still gain longer OS and a better prognosis (Vrdoljak et al., 2016; Li and Kroetz, 2018).

### Oncolytic Virus Therapy

Oncolytic virus therapy is mainly based on the infection of attenuated virus in tumor cells to kill tumor cells or boost pre-existing native immune response. This leads to a domino effect including death of infected tumor cells, destruction of tumor vessels, chained tumor antigen presentation, and further immune activation (Russell et al., 2012). The American Food and Drug Administration (FDA) has approved the immunogenic oncolytic virus as a new treatment for advanced melanoma. HSV has a large DNA-based genome which is ideal for gene modification. In a clinical case report, ECHO-7 is also available for melanoma via intranasal administration. The most effective one is the granulocyte-macrophage colony-stimulating factor (Andtbacka et al., 2015; Proboka et al., 2018). Treatment using a combination of an immune checkpoint inhibitor and the new medicine talimogene laherparepvec, the modified herpes simplex virus, shows that the oncolytic virus treatment is a breakthrough in advanced melanoma treatment (Andtbacka et al., 2015; Chesney et al., 2018). There are still plenty of impediments to the application of oncolytic virus treatment on brain metastasis. First, it is doubtable that patients enrolled, with distant metastatic lesions, obtain longer OS after treatment. Also, for brain metastasis patients, it is risky to operate intertumoral injection to all brain metastasis lesions or high-dose virus systemic delivery (Ferguson et al., 2012). Meanwhile, the blood–brain barrier is still an obstacle for drug delivery. Besides the targeting specificity to target tumor cells, interruption of the tumor microenvironment of oncolytic virus in the brain has been considered. IgM contributes to the neutralization of antibodies, and the B-cell immunosuppressive agent cyclophosphamide could partially suppress this innate antiviral response (Ikeda et al., 1999). In order to conquer the antiviral activity present in plasma and through the blood–brain barrier, Du designed the MSC-based cellular carriers to deliver the oncolytic virus to multiple brain metastasis lesions by ICA injection (Du et al., 2017). The oncolytic virus stimulated distant T cells to expand and migrate to tumor tissues, which meditate continuous immune feedback to the tumor antigen. Although the oncolytic virus induced an increase in PD-L1 expression level and the population of PD-l+ T cell, Tregs were downregulated, and a general antitumor effect is shown (Jiang et al., 2019). In summary, oncolytic virus therapy on brain metastasis still has several problems to be solved: (1) how to deliver oncolytic virus both safely and effectively; (2) how to deal with the initial immune response in plasma; and (3) how to weaken the unfavorable effect of T cells.

## Discussion

With the present clinical trials considered, it is still vague whether new treatments can provide a better life quality for brain metastasis patients. The lymphatic system in the brain has been discovered for nearly 6 years. Yet, we have not illustrated a clear image of the function of the native immune microenvironment. Lymphatic drainage of the brain does play an important role in brain metastasis treatments. The unique microenvironment of the brain and blood–brain barrier establish an immunosuppressive surrounding of tumor cells and TILs. Accumulative Tregs interrupted the function of CAR T-cell-based treatment. A similar immunosuppressive effect is also observed in native brain tumors. CAR T cells quickly shifting to an exhausted phenotype always happens along with upregulation of CTLA-4 and PD-L1. This is thought to be a native protective mechanism to balance the local activation of T cells (Peters et al., 2017; Zhou et al., 2018; Herbst et al., 2020).

Adaptive immune response might also be the main reason leading to poor clinical outcomes. In general, checkpoint inhibitor treatments like PD-1/PD-L1 blockade shows a significantly better prognosis for those patients in the early stages or with EGFR mutations. In NSCLC patients with high PD-L1 expression, the PD-L1 inhibitors is the first-line choice even for those with previously untreated metastasis (Taggart et al., 2018; Steindl et al., 2021). Consequently, there is great expectation for PD-L1 in the treatment of brain metastasis. As a matter of fact, the checkpoint inhibitor has not provided the effect that researchers expect. For brain metastasis patients using PD-L1/PD-1 inhibitor as a first-line treatment, the response rate of metastasis tumor is generally lower than that of the primary lesion. A combination of two kinds of checkpoint inhibitors might show a better response rate (Mansfield et al., 2016; Kim R. et al., 2019). A radiation sensitizer improves the central nervous system response rate and accesses a longer median OS, the effect of which is to reshape the metabolic environment of the brain metastasis tumor site (Table 1).

**TABLE 1:** A short summary of relevant clinical trials: clinical trials regarding ICP treatments and the allosteric modifier of hemoglobin that have been fully published since 2001–2021.

**Trial Identifier**	**Drugs**	**Phase**	**Tumor**	***N***	**CNS ORR**	**Median OS (month)**	**Median PFS (month)**	**Median CNS PFS (month)**
NCT02085070	Ppembrolizumab	II	NSCLC	18	33%	7.7	Not mention	Not mention
			Melanoma	18	22%	NR	Not mention	Not mention
NCT02374242	Nivolumab + ipilimumab	II	Melanoma	35	46%	NR	13.8	NR
	Nivolumab			25	20%	18.5	2.6	2.5
NCT02460068	Ipilimumab	II	Melanoma	72	Not mention	7.0	1.4	1.5
						3.7	1.2	1.2
NCT01703507	Radiotherapy + ipilimumab	I	Melanoma	26	57.7%	8.0	2.5	2.53
						10,5	2.1	2.45
NCT00623766	Ipilimumab	III	Melanoma	604	Not mention	13.5	Not mention	Not mention
						10.7		
				706	Not mention	11.4	Not mention	Not mention
						11.1		
NCT00005887	Radiotherapy + efaproxiral	III	BC, NSCLC, other	265	46% (ORR)	5.4	Not mention	Not mention
	Radiotherapy + efaproxiral	II	BC, NSCLC, other	57	35%	6.4	Not mention	Not mention

*Though the application of new treatments has made a difference to brain metastasis tumor, these treatments still have not provided satisfactory clinical outcomes. The combination of two kinds of checkpoint inhibitor might show a better response rate. A radiation sensitizer is proven to be helpful for improvement of the central nervous system response rate and access to longer median OS. NR: not reached. Not mentioned: not the primary endpoint or not researched.*

Yet, a single application of the checkpoint inhibitor doesn’t achieve a satisfactory outcome, which might be associated with tumor microenvironment adaptation (Mansfield et al., 2016; Kim R. et al., 2019). The low response rate may account for the overstimulation of immune cells in circulation. When tumors finish their journey of metastasis, the unique immune microenvironment in the brain has no choice but to continue to maintain the privilege of the immune response. The astrocyte microenvironment in brain metastasis is collectively suppressed. Astrocytes and microglia may interact with tumor cells, immune cells, and other neurons, leading to the damage of the blood–brain barrier and promoting brain metastasis (Keir et al., 2008; Moon et al., 2014; Chen et al., 2016; Cherkassky et al., 2016). Tumor-associated macrophages also tend to alter the M2 subgroup phenotype, which is associated with the suppressed immune environment. Mutation of the primary tumor enhance such effects (Peng et al., 2020; Yin et al., 2020). Therefore, the strategy of brain metastasis treatment is supposed to stimulate the suppressed immune environment and turn the “cold” tumor into “hot”. The combination of immune checkpoint inhibitors and other immunotherapies may reach better clinical outcomes (Mansfield et al., 2016; Wang et al., 2017; Kim R. et al., 2019; Zou et al., 2019).

The relationship between metabolism and tumor microenvironment has also drawn more and more attention. Metabolic feature supports tumor growth by taking up nutrients and adapting to the tumor microenvironment (Hawrylowicz and O’Garra, 2005; DeBerardinis, 2020). In response, the tumor microenvironment give feedback, having a profound influence on the treatment of brain metastasis. Theoretically, the inhibition of hypoxia, aerobic glycolysis, and glutamine supply for tumor cells can change or even end the chaotic metabolic microenvironment and turn it into a more hospitable environment to accept tumor microenvironment-targeted therapy.

The crosstalk among tumor cells, the brain–blood barrier, and cell junctions in brain metastasis is a complicated network that influences a great number of molecules and cells (Wettschureck et al., 2019; Fares et al., 2020). From tumor stimulation to immune infiltration and response, the function and dysfunction of surfaces among astrocytes, endothelial cells, and tumor cells are critical to the treatment of brain metastasis (Sampson et al., 2008; Weis, 2008; Lauko et al., 2020). Although disruption of the brain–blood barrier facilitates tumor migration, understanding the mechanism of barrier leaking is vital to drug delivery (Tang et al., 2019). The most critical obstacle is that there is no systematic and double-blind clinical trial presented. The mechanism underlying treatments targeting cell junctions remains unknown. It could be a proper supplementary for other treatment strategies.

Some anti-tumor medicines are proven to be effective for brain metastasis. The original function is well-known like targeting DNA replication, but the interaction with the tumor microenvironment is usually neglected. Topotecan, selective inhibition of topoisomerase I, disrupts the replication and transcription processes in the tumor cells, which leads to cell death. At the same time, it also inhibits HIF-1α by affecting RNA transcription and blocking the insulin-like growth factor-I (Beppu et al., 2005; Rapisarda et al., 2009). Topotecan is proven to decrease HIF-1α accumulation in the combination of bevacizumab to inhibit tumor growth in U251 hypoxia response element xenografts. As expected, the addition of topotecan to bevacizumab eliminates the negative effect of increased hypoxia (Rapisarda et al., 2009).

Overall, the regulation and balance of the tumor microenvironment in brain metastasis will be the priority consideration for combination therapy optimization in the future.

## Author Contributions

ZG, HL, and XC contributed equally to the literature analysis, integration, and manuscript writing. YZ and AL contributed to the proofreading. LJ was responsible for the project design, organization, and proofreading. All authors listed have made a substantial, direct and intellectual contribution to the work, and approved it for publication.

## Conflict of Interest

The authors declare that the research was conducted in the absence of any commercial or financial relationships that could be construed as a potential conflict of interest.

## Publisher’s Note

All claims expressed in this article are solely those of the authors and do not necessarily represent those of their affiliated organizations, or those of the publisher, the editors and the reviewers. Any product that may be evaluated in this article, or claim that may be made by its manufacturer, is not guaranteed or endorsed by the publisher.
